# Simulation-Based Neonatal Resuscitation Education for Undergraduate Anesthesia Students: A Pre- and Post-Evaluation of Knowledge and Clinical Skills

**DOI:** 10.1155/2022/7628220

**Published:** 2022-06-24

**Authors:** Debas Yaregal Melesse, Henos Enyew Ashagrie

**Affiliations:** Department of Anesthesia, College of Medicine and Health Sciences, University of Gondar, Gondar, Ethiopia

## Abstract

**Background:**

Nearly one in five hundred babies unexpectedly need resuscitation at birth, and the need for resuscitation is often unpredictable. A large majority of these deaths occur in low-resource settings and are preventable. Appropriate resuscitation techniques are crucial to the survival of newborn infants. Therefore, producing skilled health professionals in teaching institutions is mandatory to perform this activity.

**Objective:**

The study aimed a pre- and post-evaluation of knowledge and clinical skills performance of anesthesia students completing simulation-based neonatal resuscitation training at a Teaching Referral Hospital.

**Methods:**

A pre-post-intervention study was conducted on undergraduate final-year anesthesia students at Comprehensive and Specialized Teaching Referral Hospital, Ethiopia. We used a validated checklist to follow the students' performance (American Heart Association, 2005, and Ogunlesi et al., 2012). The data were collected through this checklist. The collected data were analyzed with statistical package for social sciences (SPSS) version 20. Categorical variables were analyzed with chi-square test, and a *p*-value <0.05 was considered as statistically significant.

**Results:**

A total of 51 students participated in the study. Twenty of them were females. The pre-intervention knowledge of the respondents about aspects of evaluation for neonatal resuscitation was 90.2%, and post-intervention was 94.1%; the knowledge of the respondents about aspects of appropriate actions at pre- and post-interventions was 73.4% and 83.1%, respectively. *Conclusions and recommendations*: This study showed that there was improvement of post-interventions knowledge and clinical skills of undergraduate anesthesia students for both aspects of evaluation and appropriate actions for neonatal resuscitation. We recommend that students who attached clinical anesthesia practice should take at least simulation-based training at skill laboratories timely.

## 1. Introduction

About 1 in 500 babies unexpectedly need resuscitation at birth [1]. Globally, 2.7 million newborns die each year, largely as a result of birth asphyxia and complications of preterm birth and infections [2]. A large majority of these deaths occur in low-resource settings. Continuous neonatal resuscitation training based on the local needs in resource-limited countries is essential to provide confidence in healthcare professionals, to initiate resuscitation and to improve newborn outcomes [3–5]. Approximately 5%–10% of newborns require some support to adapt to the extrauterine environment for establishment of regular respiration, and it is already known that neonatal resuscitation training (NRT) of birth attendants using mannequins results in improved knowledge and skills needed for resuscitation but needs to establish the best combination of settings, trainee characteristics, and training frequency to sustain the existing effect on perinatal mortality reduction [6]. Between 5%–10% of all babies born in facilities need some degree of resuscitation, such as tactile stimulation or airway clearing or positioning, and approximately 3%–6% require basic neonatal resuscitation, consisting of these simple initial steps and assisted ventilation [7]. A study showed that the overall incidence of asphyxia of the newborn was 22.9% in Africa [8]. The World Health Organization (WHO) estimates that 99% of the 3.8 million neonatal deaths occur in developing countries [9].

The neonatal resuscitation team may consist of nurses, family physicians, midwives, pediatricians, obstetricians, anesthetists, and respiratory therapists [10]. Simulation-based training (SBT) largely bases on appropriate implementation of system integration [11]. A study involving nursing and midwifery students reported that students scored 38%, 49%, 20%, and 72% of the total score in the initial steps of resuscitation, positive pressure ventilation, intubation, and chest compression, respectively [12].

An observational study found six errors during resuscitation that include deep suctioning, excessive stimulation, not communicating the heart rate, and not reevaluating bag valve mask ventilation [13]. Even though advances in training on neonatal resuscitation, neonatal mortality rate has not been significantly reduced yet [14]. The study conducted on nurses and midwives in Eastern Ethiopia showed that study participants had low knowledge of basic neonatal resuscitation [15]. A review done by Johnson PA and Schmölzer GM concluded that ineffective heart rate (HR) assessment significantly increases the risk of hypoxic injury and infant mortality [16].

A significant shift toward more rationale resuscitation practices was indicated by a decline in the use of chest compression and medication (*p* < 0.001 for each), and an increase in the use of bag and mask ventilation [17]. Knowledge retention often does not decline at the rate that practical skill retention declines, indicating that birth attendants need continuous refresher training that focus on practical skills [18].

A brief review highlights the usefulness of simulation-based training (SBT) as an alternative, feasible, and flexible design for education amid the pandemic COVID-19 [19]. The combination of resuscitation science, strategies to increase educational effectiveness, and implementation of interventions with high coverage and quality has resulted in reduced rates of asphyxia-related neonatal mortality [20]. Therefore, we aimed to a pre- and post-evaluation of knowledge and clinical skills performance of anesthesia students completing simulation-based neonatal resuscitation training at a Teaching Referral Hospital in new setting and new trainee characteristics.

## 2. Rationale

Our undergraduate final-year students attended several (100–150) births according to their log book record. However, there are still some deficits, particularly on the decision to start the resuscitation and on following internationally recommended guidelines. This has been evidenced by the fact that practical skill retention declines at a higher rate than knowledge retention, and birth attendants often face challenges with the application of their skills during real scenarios [18]. Neonatal deaths occur during delivery because of lack of proper resuscitation management. This situation is mostly associated with the skill gap of health professionals. Globally, neonatal death, especially in developing countries, is a serious problem. The quality of health services facilities and skill gap of health professionals are some of the reasons for it. In Ethiopia, healthcare provider students' performance abilities have its own problems during the management of neonatal asphyxia. Therefore, simulation-based training for healthcare professional students will be one of the solution/reduction strategies for the root cause of the problem.

## 3. Objective

The study aimed a pre- and post-evaluation of knowledge and clinical skills performance of anesthesia students completing simulation-based neonatal resuscitation training at a Teaching Referral Hospital in Ethiopia, 2022.

## 4. Methods

### 4.1. Study Design and Period

A pre- and post-interventional study was conducted to answer a question: “do undergraduate final-year anesthesia students show improvement on their knowledge and clinical skills through simulation-based education?” based on the hypothesis that simulation-based education for neonatal resuscitation has no effect on knowledge and clinical skills of final-year anesthesia students at a Comprehensive Specialized Teaching Referral Hospital in Ethiopia from December 25, 2021-January 10, 2022 G.C.

### 4.2. Study Population

All undergraduate final-year anesthesia students at a Comprehensive Specialized Teaching Referral Hospital in Ethiopia, 2021/2022 G.C., were included in this study.

### 4.3. Inclusion and Exclusion Criteria

#### 4.3.1. Inclusion Criteria

All undergraduate final-year anesthesia students at a Comprehensive Specialized Teaching Referral Hospital in Ethiopia, 2021/2022 G.C., who attended the full training days voluntarily were included in the study.

#### 4.3.2. Exclusion Criteria

Undergraduate final-year anesthesia students who were not available during the training period and other categories of students were excluded.

### 4.4. Sample Size and Sampling Technique

A survey, in all undergraduate final-year anesthesia students who participated in simulation-based training for neonatal resuscitation education, was conducted, and the data were collected with convenience sampling technique. Fifty-one undergraduate final-year anesthesia students participated in the study.

### 4.5. Compliance with Ethical Standards

This study was approved by Institutional Review Board (IRB) of the University of Gondar, School of Medicine, with reference number SoM/02/2021. Informed consent was obtained from each participant.

### 4.6. Data Collection Procedures and Analysis

We used a structured and validated checklist to assess the students' knowledge and clinical skills [21]. It contained 20 simple statements arranged in two sections.  Section 1 tested the evaluation and identification of babies requiring assistance, while Section 2 tested appropriate decisions and actions such as appropriate warming, stimulation, airway clearance, and ventilation techniques.

The answers were “Yes,” “No,” or “Do not know.” Each practice performed/answered correctly, earned 1, incorrect, and do not know earned no mark. A minimum score of 75% defined adequate knowledge and skills in each section and for the overall intervention. After assessing with these two sections of the questionnaire and identifying the level of knowledge and clinical skills of the students, we gave 17-day simulation-based training at the skill laboratory. After the training, we reassessed the students' knowledge and clinical skills improvement with similar tools. Descriptive statistics were conducted, and the results were presented as text, frequency, and percentages. Categorical variables were analyzed with chi-square test, and *p*-value <0.05 was considered as statistically significant.

## 5. Results

Fifty-one students were involved in this study. Twenty of them were females, and 31 were males. Ninety-six percent of the participants attended spontaneous vaginal births, and four students had never attended any spontaneous birth yet. Students had better knowledge for evaluation than for appropriate actions (90.2% V 73.4%) for neonatal resuscitation during pre-intervention time. Before the intervention, our students were specifically poor in following the correct sequences during the resuscitation, in which 36.4% of the participants failed to act according to the guideline recommendations. It was surprising that the majority (82.4%) of the students did not decide the need of resuscitation for a neonate born with meconium stained liquor (Table 1); otherwise, all of them achieved adequate knowledge on the evaluation section compared with the cutoff point. 19.6% of the students had poor practical skill on coordinating ventilation to chest compression ratio (Table 2). There were identified gaps from the students in knowledge and clinical skills on assembling the required equipment necessary for neonatal resuscitation. 90.2% of the students had adequate knowledge (scored ≥75%) for evaluation, and 73.4% of the students had adequate knowledge (scored ≥75%) for aspects of appropriate actions for newborn resuscitation.

As shown in the pre-intervention section, our students had gaps on evaluation and appropriate actions for neonatal resuscitation. The outcome of a compromised fetus depends on early detection of the problem and immediate reaction. The procedure should be conducted according to the sequence of internationally accepted guidelines in 100% adherence rate [21]. However, 36.6% of the study participants failed to decide and act according to these accepted guideline recommendations before the intervention. Therefore, to improve our students' knowledge and performance, it seems that we shall design a simulation-based training before graduation. After identifying the gaps, we designed a well-organized 17-day simulation-based training for undergraduate final-year anesthesia students of 2021/22. Two senior anesthetists were invited for delivering the theoretical aspect of neonatal resuscitation and demonstration with manikins (hands-on simulation) based on the recommendations of the studies and guidelines [21–23] at the class room and skill laboratory, respectively, from December 25, 2021-January 10, 2022 (Table 3). The hands-on simulation sessions used a trainer-trainee ratio of 1:5 at skill laboratory in two stations. The same content of the neonatal training with simulators/manikins was delivered to the same 51 undergraduate final-year anesthesia students. All materials were provided in English, including course materials, handouts, and checklists.

The performance of the intervention met its target according to our plan. First, we identified the current knowledge and practical deficit of the students with a validated checklist, and then we planned the way how to fill this gap because the inadequate knowledge and inappropriate attitudes of healthcare providers toward neonatal resuscitations were well identified by different researchers. We carefully evaluated the real knowledge and skill gap of the students. We communicated with each other the way to manage and plan the intervention (simulation-based education/training). In the plan, we selected the time and place to perform an intervention. All students performed the demonstration according to the curriculum. Therefore, the plan was hit its target. In the direct observation session, all of them tried to practice the way how to manage neonatal resuscitation (days 13–17). Our plan was focused on improving the students' knowledge and performance for newborn resuscitation. Thus, their knowledge and clinical skills were reassessed with similar checklists that were used in pre-intervention **(**Tables 4 and 5). Although the majority of them were following the procedures, still some of them require further time and attachment to adhere with the recommendations.

## 6. Discussion

This study aimed to evaluate undergraduate final-year anesthesia students' knowledge and clinical skills improvement through simulation-based training for neonatal resuscitation education with a pre- and post-intervention study. The students' gaps were assessed and reassessed at pre- and post-intervention period, respectively, with similar validated checklist. The results showed that there were improvements after the intervention in both aspects of evaluation for neonatal resuscitation and appropriate decisions and actions (46 (90.2%) Vs. 48 (94.1%)) and (37 (73.4%) Vs. 42 (83.1%)), respectively. The current study was consistent with a study conducted by Murphy AA and Halamek LP which recommends that simulation-based education for medical students plays a paramount role for their clinical practice [24]. This might be because both studies used manikins as a simulator (similar level fidelity). Our study is also consistent with a study conducted in the resource-limited setting, Zanzibar, that showed improvement of healthcare professionals' knowledge and skills through simulation-based neonatal resuscitation training [3]. This might be the fact that we used the same study design (a pre-post-intervention study) and we both used the same assessment tool (American Heart Association Guidelines) to assess knowledge and clinical skills for neonatal resuscitation among healthcare workers.

In this study, 90.4% of the study participants had adequate knowledge to evaluation for neonatal resuscitation. This study was not similar with a study conducted in Eastern Ethiopia entitled, “Knowledge of Basic Neonatal Resuscitation and Associated Factors Among Midwives and Nurses in Public Health Institutions in Eastern Ethiopia” [15]. The discrepancy could be reasoned out that the study participants in this study were students who were taking the course in their curriculum acutely and the later study participants were professionals who had other tasks other than education/training like leading families.

The current study showed an improvement of students' knowledge to appropriate decisions and actions for neonatal resuscitation from 37 (73.4%) to 42 (83.1%) after simulation-based neonatal resuscitation education. This study is in line with a study conducted by A. K. Deorari et al. which showed that significant shift toward more rational resuscitation practices was indicated by a decline in the use of chest compression and medication (*p* < 0.001 for each), and an increase in the use of bag and mask ventilation (*p* < 0.001) [17]. This might be the fact that impact of simulation-based education and practices leads to perfection.

Our results showed substantial improvements in each item of the checklist, even though insignificant statistically. This statistically insignificant results are inlined with the study that showed subsequent training in the Neonatal Resuscitation Program which did not significantly reduce the neonatal mortality rates, although the rate of stillbirths was reduced [25].

This study failed to show consistency with the guidelines and previous studies which recommend that healthcare providers should be fully compliant (100%) with the standards [25–28]. The discrepancy might be the lack of exposure of students to guidelines and laboring parturients.

In the current study, there were significant improvements on the following aspects of appropriate decisions and actions for neonatal resuscitation: determination of APGAR score is the first step in neonatal resuscitation, hydrocortisone injection is important in neonatal resuscitation, one pre-warmed towel is adequate for neonatal resuscitation, and when co-coordinating ventilation and chest compression one session of ventilation is done after every 3 chest compressions (*p* < 0.001). These findings were similar with studies conducted in neonatal resuscitation training with simulators [6, 7, 13, 17, 18, 22, 24, 27, 29, 30]. The scientific explanation behind it was the current study, and all these studies advocated that healthcare providers and students should focus their education on areas of theoretical background plus skill laboratory training with simulators specially with high fidelity simulators. Generally, this study showed improvement of student performance but low compared to guideline recommendations that should be adhered with internationally accepted guidelines [21].

## 7. Limitations of the Study

Our paper has many limitations. One of the limitations of this study was the lack of high fidelity simulator as the setting is low resourced. Small sample size, lack of independent variables, effective-controls, and rigor were also limitations of the study.

## 8. Conclusions and Recommendations

This study showed that there was significant improvement of post-intervention knowledge and practice of undergraduate graduating class anesthesia students for both aspects of evaluation and appropriate actions for neonatal resuscitation. We recommend that students who attached clinical anesthesia practice should take at least simulation-based training at skill laboratories timely.

Hence, future research should focus on the short-term and long-term effectiveness of a neonatal resuscitation training/education, including the health outcomes of newborns. Ideally, these studies need to include factors that influence infant mortality, such as prematurity, sepsis, birth asphyxia, and malnutrition. Collaborations between pediatric healthcare professionals and governmental support to improve the competencies of neonatal resuscitation performance will benefit the medical staff, infants, and parents, through both a neonatal resuscitation training and training of neonatal health promotion and management. These combined efforts might contribute to reaching the “Sustainable Development Goals in reducing neonatal mortality to at least 12 per 1,000 live births.”

## Figures and Tables

**Table 1:** Pre-intervention knowledge of evaluation in neonatal resuscitation of undergraduate final-year anesthesia students at a comprehensive specialized teaching referral hospital in Ethiopia, (*N* = 51).

S. no	Item	Correct *n*, (%)	Incorrect *n*, (%)
1	Meconium stained liquor does not suggest that the newborn may require neonatal resuscitation*∗*	9 (17.6)	42 (82.4)
2	Mucus extractor and infant Ambu-bags are not always required in the delivery room*∗*	2 (4)	49 (96)
3	Respiratory effort, color, and heart rate (cord pulsation) are used to decide if a newborn infant requires resuscitation	46 (90.2)	5 (9.8)
4	After warming, sucking, and drying, if an infant remains apneic, the least important step is to Ambu-bag*∗*	8 (15.7)	43 (84.3)
5	After delivery, it is important to ascertain that the heart rate is above 100/minute	48 (94.1)	3 (5.9)
6	Cyanosis and heart rate less than 100/minute are danger signs in newborn infants	48 (94.1)	3 (5.9)

*N* = total number of students; *n* = number of students who answered “correct” or “incorrect”; *∗*negatively framed statements.

**Table 2:** Pre-intervention knowledge of appropriate decisions and actions in neonatal resuscitation of undergraduate final-year anesthesia students (*N* = 51).

S. no	Items	Correct *n*, (%)	Incorrect *n*, (%)
1.	The correct order of initial resuscitation measures includes keeping warm, sucking, drying, and stimulation	35 (68.6)	16 (31.4)
2.	Determination of APGAR score is the first step in neonatal resuscitation*∗*	20 (39.2)	31 (60.8)
3.	The first step in neonatal resuscitation is warming and the infant up	32 (62.7)	19 (37.3)
4.	Hydrocortisone injection is important in neonatal resuscitation*∗*	14 (29.5)	37 (72.5)
5.	One pre-warmed towel is adequate for neonatal resuscitation*∗*	21 (41.2)	30 (58.8)
6.	The nose of the newborn infant should be suctioned before the mouth*∗*	24 (47.1)	27 (52.9)
7.	The Ambu-bag appropriate for the newborn must cover the nose, mouth, and chin	48 (94.1)	3 (5.9)
8.	Suctioning of the airways should be continuous when secretions in the airway are “excessive”*∗*	6 (11.8)	45 (88.2)
9.	Chest compression must be accompanied by Ambu-bagging	46 (90.2)	5 (9.8)
10.	Holding aloft and slapping the buttocks is an acceptable way to stimulate an apneic baby*∗*	18 (35.3)	33 (64.7)
11.	The best way to assess the success of Ambu-bagging is to observe a rise of a fall in the chest wall	45 (88.2)	6 (11.8)
12.	Exposure to heat may stimulate a newborn baby who has apnea *∗*	10 (19.6)	41(80.4)
13.	During Ambu-bagging, breaths should be delivered at a rate of 40–60/minute	33 (64.7)	18 (35.3)
14.	When co-coordinating ventilation and chest compression, one session of ventilation is done after every 3 chest compressions	41(80.4)	10 (19.6)

*N* = total number of students; *n* = number of students who answered “correct” or “incorrect”; ^∗^negatively framed statements.

**Table 3:** Content/curriculum of simulation-based neonatal resuscitation training for undergraduate final-year anesthesia students at a comprehensive specialized teaching referral hospital in Ethiopia.

Date	Time	Title	Content
*Day 1*	9:00–10:00 am	Highlighting the principles of newborn resuscitation in the class room	Introducing the 2005 American Heart Association (AHA) Guidelines for Cardiopulmonary Resuscitation (CPR) and Emergency Cardiovascular Care (ECC) of Pediatric and Neonatal Patients: Pediatric Basic Life Support by senior anesthetist (for all 51 students)
	10:00–11:00 am	Indications of neonatal resuscitation in theory class room	Delivering the theoretical aspects about the indications of neonatal resuscitation such as asphyxia, meconium stained, and APGAR score with power point by one of the trainers (for all 51 students)
	11:00–12:00 am	Evaluation of newborns for resuscitation in the class room	Power point presentation about the necessary systems and its signs for evaluation of a newborn for resuscitation for all 51 students
	2:00–5:00 pm	Necessary equipment and medications for neonatal resuscitations (theory class room)	Necessary equipment like Ambu-bag, facemask, laryngeal mask airway, tracheal tubes, etc., and medications like adrenaline, hydrocortisone, bicarbonate, etc., were presented by the trainer for all 51 students

*Day 2*	9:00–10:00 am	Initial steps of neonatal resuscitation (class room)	The correct order of resuscitation like providing warmth, giving the right position, clearing the airway, drying the baby, and stimulating breathing was described
	10:00–11:00 am	Principles of chest compression to ventilation in theory class room	A brief description of indications of compression, procedures, necessary devices, contraindications and indications of ventilation (mechanical or assisted), and compression/ventilation ratio
	11:00–12:00 am	Techniques of stimulation and handling of newborns (theory class room)	Holding aloft and buttock stimulation are not recommended during resuscitation (brief description of principles of stimulation by trainers for all 51 participants)
	2:00–5:00 pm	Endotracheal intubation and Ambu-bag usage	The indications, devices, procedures, and contradictions of intubation and principles of Ambu-bagging are well presented by the trainers for all 51 students/participants

*Day 3*	9:00–10:00 am	Neonatal resuscitation evaluation (hands-on simulation) at skill laboratory	All 51 students were participated in evaluation of neonatal resuscitation (rapid evaluation) in 2 stations
	10:00–11:00 am	Neonatal resuscitation (hands-on simulation) at skill laboratory	Divided into 2 groups for 1 and 2 in 2 stations, each group had 1 trainer and 5–6 students, giving the scenario training followed by initial step to intubation
	11:00–12:00 am	Use of resuscitation devices (hands-on simulation) at skill laboratory	The indication for starting mechanical ventilation, initial breaths, assisted ventilation, and end-expiratory pressure
	2:00–5:00 pm	Techniques of Ambu-bagging	All students divided into 2 like A and B and did Ambu-bagging following the trainer in each station (station 1 and 2)

*Day 4*	9:00–10:00 am	Chest compression (hands-on simulation)	The indications, devices, procedures, and contradictions of chest compression and the trainer showed to the students the technique
	10:00 am–5:00 pm	Ambu-bagging technique and effectiveness (hands-on simulation) at skill laboratory	The trainers in each station showed the technique to the students

*Day 5–17*	From 9:00 am–5:00 pm	Hands-on simulation with manikins in skill laboratory by themselves	Students divided into 2 two groups; 5 or 6 each with one senior anesthetist (trainer) and assigned into 2 stations and practice each step necessary for neonatal resuscitation under supervision

Nota bene: the maximum time allowed for each group was 12 minutes considering 1:5 ratio for 51 students in two stations in 1-hour period.

**Table 4:** Comparison of pre-intervention and post-intervention knowledge on evaluation in neonatal resuscitation of undergraduate final-year anesthesia students (*N* = 51).

S. no	Students' performance				
	Items	Pre-intervention, *n* (%)	Post-intervention, *n* (%)	Improvement rate (%)	*p*-value
1	Meconium stained liquor does not suggest that the newborn may require neonatal resuscitation	42 (82.4)	43 (84.3)	1.9	0.75
2	Mucus extractor and infant Ambu-bags are not always required in the delivery room	49 (96)	51 (100)	4	0.54
3	Respiratory effort, color, and heart rate (cord pulsation) are used to decide if a newborn infant requires resuscitation	46 (90.2)	46 (90.2)	0	0.99
4	After warming, sucking, and drying, if an infant remains apneic, the least important step is to Ambu-bag	43 (84.3)	48 (94.1)	9.8	0.34
5	After delivery, it is important to ascertain that the heart rate is above 100/minute	48 (94.1)	51 (100)	5.9	0.37
6	Cyanosis and heart rate less than 100/minute are danger signs in newborn infants	48 (94.1)	49(96)	1.9	0.75
7	Number of students who had adequate knowledge (scored ≥75%)	46 (90.2)	48 (94.1)	3.9	0.54

*N* = total number of students; *n* = number of students who answered the given questions “correctly.”

**Table 5:** Comparison of pre-intervention and post-intervention knowledge of appropriate decisions and actions for neonatal resuscitation of undergraduate final-year anesthesia students (*N* = 51).

S. no	Items	Students' performance			
		Pre-intervention, *n* (%)	Post-intervention, *n* (%)	Improvement rate (%)	*p*-value
1	The correct order of initial resuscitation measures includes keeping warm, sucking, drying, and stimulation	**35(68.6)**	**37(72.5)**	**3.9**	**0.54**
2	Determination of APGAR score is the first step in neonatal resuscitation	**31(60.8)**	**41(78.4))**	**17.6**	**0.001**
3	The first step in neonatal resuscitation is warming and the infant up	**32(62.7)**	**33(64.7)**	**2**	**0.75**
4	Hydrocortisone injection is important in neonatal resuscitation	**30(58.8)**	**40(78.4)**	**19.6**	**0.001**
5	One pre-warmed towel is adequate for neonatal resuscitation	**30(58.8)**	**49(96.1)**	**37.3**	**<0.001**
6	The nose of the newborn infant should be suctioned before the mouth	**27(52.9)**	**28 (54.9)**	**2**	**0.75**
7	The Ambu-bag appropriate for the newborn must cover the nose, mouth, and chin	**48(94.1)**	**51(100)**	**5.9**	**0.54**
8	Suctioning of the airways should be continuous when secretions in the airway are “excessive”	**45(88.2)**	**45(88.2)**	**0**	**0.99**
9	Chest compression must be accompanied by Ambu-bagging	**46(90.2)**	**51(100)**	**9.8**	**0.34**
10	Holding aloft and slapping the buttocks is an acceptable way to stimulate an apneic baby	**33(64.7)**	**39(76.5)**	**11.8**	**0.10**
11	The best way to assess the success of Ambu-bagging is to observe a rise of a fall in the chest wall	**45(88.2)**	**51(100)**	**11.8**	**0.10**
12	Exposure to heat may stimulate a newborn baby who has apnea	**41(80.4)**	**41(80.4)**	**0**	**0.99**
13	During Ambu-bagging, breaths should be delivered at a rate of 40–60/minute	**33(64.7)**	**37(72.5)**	**7.8**	**0.3**
14	When co-coordinating ventilation and chest compression, one session of ventilation is done after every 3 chest compressions	**41(80.4)**	**51(100)**	**19.6**	**0.001**
15.	Number of students who had adequate knowledge (scored ≥75%) on appropriate decisions and actions in neonatal resuscitation	**37 (73.4)**	**42 (83.1)**	**9.7**	**0.32**

*N* = total number of students; *n* = number of students who answered the given questions “correctly.”

## Data Availability

The data used to support the findings of the study can be obtained from the corresponding author upon request.
